# Personal, Social and Vocational Outcomes of Inclusive University for People With Intellectual Disability

**DOI:** 10.1111/jir.70042

**Published:** 2025-09-28

**Authors:** Mary‐Ann O'Donovan, Nikki Wedgwood, Greta Westermann, Fiona Rillotta, Tess Aitken

**Affiliations:** ^1^ Centre for Disability Studies, The University of Sydney, Susan Wakil Health Building—D18, Level 7, Western Avenue The University of Sydney Sydney New South Wales Australia; ^2^ Sydney School of Health Sciences, Faculty of Medicine and Health University of Sydney Sydney Australia; ^3^ Centre for Disability Studies The University of Sydney Sydney Australia; ^4^ Disability and Community Inclusion, College of Nursing and Health Sciences Flinders University Adelaide South Australia Australia; ^5^ Medicine and Health Team, Academic Services The University of Sydney Library The University of Sydney Sydney Australia

**Keywords:** college, higher education, inclusive education, intellectual disability, outcomes, postsecondary education, university

## Abstract

**Background:**

Though people with intellectual disability have historically been excluded from university education, over the past three decades programmes facilitating the inclusion of people with intellectual disability in higher education, including university, have been developed in some jurisdictions such as the United States, Canada, Ireland, Europe and Australia. There is limited examination of the full range of potential outcomes for students with intellectual disability participating in such programmes. Such research rarely explores barriers and facilitators in relation to particular programme models and the achievement of the potential outcomes. The current study set out to examine the range of outcomes associated with inclusive university programmes, with a particular focus on autonomy, self‐confidence, inclusion and employment.

**Method:**

For this scoping review, six academic databases were searched using broad search terms. Forty‐five studies meeting our inclusion criteria were subjected to quality assessment to ensure only high‐quality evidence informed data analysis. Twenty‐two studies were included in the final review, from which data were extracted to answer our research question.

**Results:**

Students were reported to achieve outcomes related to employment, confidence, autonomy and social skills. Factors reported as either facilitating or impeding positive personal, vocational and/or socio‐emotional outcomes for students with intellectual disability participating in inclusive university programmes included accessibility, expectations, supports, attitudes and acceptance, pedagogy and programme design.

**Conclusions:**

Programmes that provide individualised support for the inclusion of people with intellectual disability in university life led to primarily positive outcomes for people with intellectual disability. We argue that a whole‐of‐university approach to supporting full and genuine inclusion of students with intellectual disability is needed and we provide recommendations for research and practice.

## 1 Introduction

Going to university has become a typical part of the transition to adulthood for increasing numbers of young people. This trend is a corollary of the massification of higher education, which has raised societal expectations for greater inclusion and opportunity for traditionally under‐represented groups, such as people from lower socio‐economic groups, ethnic minorities, first in family to attend university and people with disability (Hornsby and Osman 2014; Denice 2019). Raising of expectations is occurring for people with intellectual disability also, as they wish to follow in the footsteps of their non‐disabled siblings and classmates who are increasingly heading off to university after secondary school (Turnbull et al. 2024).

Rising expectations for people with intellectual disability to gain access to higher education have resulted in some progress over the past three decades in jurisdictions such as the United States, Canada, Ireland, Europe and Australia (Grigal et al. 2022; Beschen 2018; Kubiak et al. 2019; Stefánsdóttir and Björnsdóttir 2016; Gadow and MacDonald 2019). In the United States, an estimated 6000 students with intellectual disability are enrolled in over 310 higher education institutions (Grigal et al. 2021). Yet, in Australia, only two universities have inclusive programmes; thus, progress is patchy. Moreover, people with intellectual disability are still much less likely to progress to university than their peers (Morningstar and Shoemaker 2018). Pathways to higher education broadly, and university specifically, for people with intellectual disability have been, and continue to be, fraught with barriers at a systemic, policy and societal level (Wedgwood et al. 2023). Without supported pathways and inclusion, people with intellectual disability encounter a range of barriers to accessing university due to a range of structural, institutional and interpersonal disablism.

Institutional barriers to inclusive university for people with disability generally fall into three broad areas of barriers to inclusion: organisational, attitudinal and knowledge (Fernández‐Batanero et al. 2022). For people with intellectual disability in particular, the focus of universities on intellectual pursuits and achievements can foster a culture fundamentally exclusive of intellectual disability. Other barriers for students with intellectual and developmental disabilities include transportation, lack of knowledge, lack of options, absence of personalised supports and not being integrated on the campus (Lee and Taylor 2022).

Another common barrier is that people with disability tend to leave school earlier than their peers without disability (US National School Boards Association 2019; European Agency for Special Needs and Inclusive Education 2016; Zablocki and Krezmien 2013). This is because they are often subject to bullying (Carter and Spencer 2006) and a lack of appropriate supports in school (Hong 2015). They also experience limited inclusion in mainstream schooling (Buchner et al. 2021) in addition to limited postsecondary school options (Yamamoto et al. 2014; Uditsky et al. 2012). There is also a greater likelihood of low self‐esteem among people living with disability (Nosek et al. 2003), which can be linked to social stigma and acceptance (Jung et al. 2022). These factors are underpinned by persistent low expectations of progression from others (Garrison‐Wade and Lehmann 2009; Sanders 2006) that inhibit the aspirations of students and families.

As a result, it is not uncommon for typical transitions, including postsecondary transitions, to be delayed or postponed for people with disability (Priestley 2000). Delays in life‐phase transitions are exacerbated even more so for people with intellectual disability (Glidden et al. 2012; Gray et al. 2014). This is why many university inclusion programmes attract older people with intellectual disability seeking opportunities as a more desirable alternative to supported employment or day services (Rillotta et al. 2020).

Despite the barriers created by persistent structural, institutional and interpersonal disablism, progression to higher education can be supported through early transition planning, input from guidance counsellors and inclusion of the student and family in transition planning (Bell et al. 2017). Attending university also requires well‐structured supports at university. Indeed, specialised models of university inclusion are essential to ensuring equity of higher education for people with intellectual disability.

Three alternative models for people with intellectual disability and autism to transition to higher education have been identified (Hart and Grigal 2009; Hart et al. 2006). Known as substantially separate, hybrid and fully inclusive, these models differ with regard to the level of integration with the general student population, the nature of enrolment and the nature of supports provided. Though the authors are aware that increasingly, this categorisation is not being used in the United States, it continues to be used in other jurisdictions (Kubiak 2015).

As the name suggests, the substantially separate model refers to programmes in which the involvement of students with intellectual disability in university life is substantially separate from that of the general student population, for example, in segregated classes. The hybrid model refers to programmes in which students with intellectual disability participate in a mix of both segregated and integrated university activities (Kubiak 2015). Fully inclusive programmes are based on the ‘inclusive individualized support model’, of inclusion in which students with intellectual disability are provided the individualised services and supports necessary for inclusion in university alongside the general student body (Hart et al. 2004, 57). Many fully inclusive programmes offer individualised support for participation in both academic and non‐academic aspects of university life and/or pathways to employment/internships (Plotner et al. 2019). Most fully inclusive programmes involve students auditing classes without gaining credit towards recognised academic qualifications (Gadow and MacDonald 2019). However, a small number offer accredited pathways to the completion of degrees (High and Robinson 2021) or other academic qualifications (O'Donovan 2021; Kubiak 2015).

Participating in a transition, which is part of the normative life course—such as postsecondary education—holds value in and of itself but has also been shown to have a positive impact on health, identity development, social connections and employment outcomes for all young people, with or without disability (Hart et al. 2010; Hornsby and Osman 2014). Thus, it is unsurprising that research shows that participation in any type of higher education—not just university—is beneficial for people with intellectual disability (Lee and Taylor 2022). For instance, positive outcomes for students with intellectual and developmental disabilities who take part in various types of postsecondary education include improvements in health, adaptive behaviour, employment opportunities and independent living (Aron and Loprest 2012; Lee and Taylor 2022). Participation in inclusive university programmes more specifically has also been shown to have a wide range of positive outcomes for students with intellectual disability. Alongside increased learning and employment skills, going to university helps people with intellectual disability develop self‐determination, self‐confidence, social connections and a sense of belonging as a valued member of a community that traditionally excluded them (O'Brien et al. 2018).

Despite a substantial body of evidence of a wide range of social, vocational and personal benefits of inclusion at university for people with intellectual disability, we currently have an incomplete picture of the full range of potential outcomes. To begin with, studies of inclusive university programmes for students with a range of disabilities often conflate the outcomes for students with autism, learning disabilities, ADHD and/or intellectual disabilities. Of studies which do focus on programmes exclusively for students with intellectual disability, many investigate only one or two specific outcomes, most often employment outcomes. Certainly, employment is a particularly important outcome, given people with intellectual disability are less likely than their peers without intellectual disability to be in employment (van der Zwan and de Beer 2021). In addition, there is often no distinction made between the type of programmes, with inclusive, segregated and mixed models reported together. Therefore, in order to inform best practice globally, it is important to understand the range of outcomes achieved from specific programme types, as well as the barriers to, and facilitators of, the full range of personal, vocational and socio‐emotional outcomes of inclusive university for people with intellectual disability.

Clearly, a stronger evidence‐base is needed to identify a continuum of best practice in the inclusion of people with intellectual disability at university. To this end, our review examines international peer‐reviewed literature on inclusive university programmes to identify the full range of outcomes for students with intellectual disability. Our focus was on 4‐year academic institutions where inclusion programmes for people with intellectual disability are less common than in other postsecondary institutions, like community colleges. Specifically, the authors were interested in programmes that fell within the ‘totally inclusive’ definition or ‘the hybrid model’ as defined above. Our review question was:

What are the range of outcomes associated with inclusive university including autonomy, self‐confidence, inclusion and employment; and what factors support or hinder achievement of these outcomes for students with intellectual disability?

## 2 Methods

Our goal was to focus on contemporary research studies because they are potentially more likely to align with the goal of promoting quality of life, as opposed to a deficit model of disability, more common in older studies. Earlier articles on inclusive programmes were also more likely to describe programmes than to explore student experiences or evaluate student outcomes (Whirley et al. 2020). We therefore limited the scope of our review to literature published in the past two decades. This enabled us to conduct a rapid review (Khangura et al. 2012; Tricco et al. 2015).

The inclusion criteria for the review were peer‐reviewed journal articles written in English, which

- were published within the last 20 years (2000–2021);
- described a research population including people with intellectual/developmental disability or impairment, Down syndrome, ‘mental retardation’ or learning disability where it refers to intellectual disability (such as in a UK context). As inclusive university models designed specifically for people with intellectual disability are not typically limited to transition‐age students, the current scoping review is not limited to transition‐aged students with intellectual disability;
- described the research setting as higher, tertiary or postsecondary education at universities or colleges;
- described programmes that fell within the ‘totally inclusive’ model or ‘the hybrid model’ where the hybrid model included some integrated classroom activity with other students and the inclusive aspect was discussed in relation to outcomes.
- described outcomes for students/participants with ID including autonomy, self‐confidence, social inclusion, employment or social skills and/or;
- presented data from multiple voices/perspectives, (e.g., students with ID, parents, teachers, mentors, student peers and programme staff), as long as student voice and/or experiences/outcomes were also captured.

The exclusion criteria for the review were journal articles which

- were published before 2000 due to focus on the most recent evidence;
- were not peer‐reviewed;
- were published in languages other than English;
- focus on people with disability, which is not intellectual unless there is co‐occurring intellectual disability (and only if data from the intellectual disability population can be disaggregated);
- describe the research setting as technical or vocational education at community colleges, technical colleges or any other adult education programmes (e.g., literacy programmes);
- do not describe outcomes related to autonomy, self‐confidence, social inclusion, social skills or employment, or which describe impacts/effects on any stakeholder groups other than the students with intellectual disability (e.g., family, teachers and non‐disabled students);
- grey literature, media items, autobiographies, biographies, theses/dissertations, review studies (the individual papers can be included if they meet criteria), study protocols, conference abstracts, books and chapters (except a small number of handpicked books and chapters).
- do not capture voices/perspectives of, or outcomes for, students with intellectual disability.
- focus only on segregated models and do not report on inclusive aspects of programmes.

### 2.1 Search Strategy

A broad search strategy was employed to retrieve relevant articles across six academic databases for the period 1 January 2000 to 10 November 2021. Reference checking and hand searching were undertaken of included studies to ensure no relevant studies were missed. The databases searched were

- Medline via Ovid
- CINAHL Complete via EBSCO
- ERIC via Ovid
- PsycInfo via Ovid
- Scopus
- Web of Science

Scoping searches were run to identify relevant search terms. The research team discussed and agreed upon a search strategy combining three groups of search terms, each containing a combination of subject headings and keywords. The first group comprised search terms for intellectual disabilities, focusing on developmental disabilities and excluding learning disabilities such as dyslexia. The second group was made up of terms for higher education institutions, with the intention of finding programmes in universities tailored to the needs of students with intellectual disability. The third group consisted of terms for outcomes of these programmes including autonomy, self‐confidence, social inclusion, social skills and/or employment.

Terms within each group were combined with the Boolean operator OR, and then all three groups were combined using AND. The search strategy was translated to suit interface differences and differences in controlled vocabularies in each database searched. A sample search strategy used in the Scopus database is presented in Table 1.

**TABLE 1:** Sample search string.

(TITLE‐ABS‐KEY (“intellectual* disab*” OR “intellectual* impair*” OR “mental* retard*” OR “development* disab*” OR “down* syndrome” OR “learning disab*”) AND TITLE‐ABS‐KEY (“higher education” OR universit* OR college* OR “tertiary education” OR “post secondary” OR undergraduate*) AND TITLE‐ABS‐KEY (autonomy OR confiden* OR inclusion OR employ* OR sociali?ation OR “self concept”))

**FIGURE 1:**
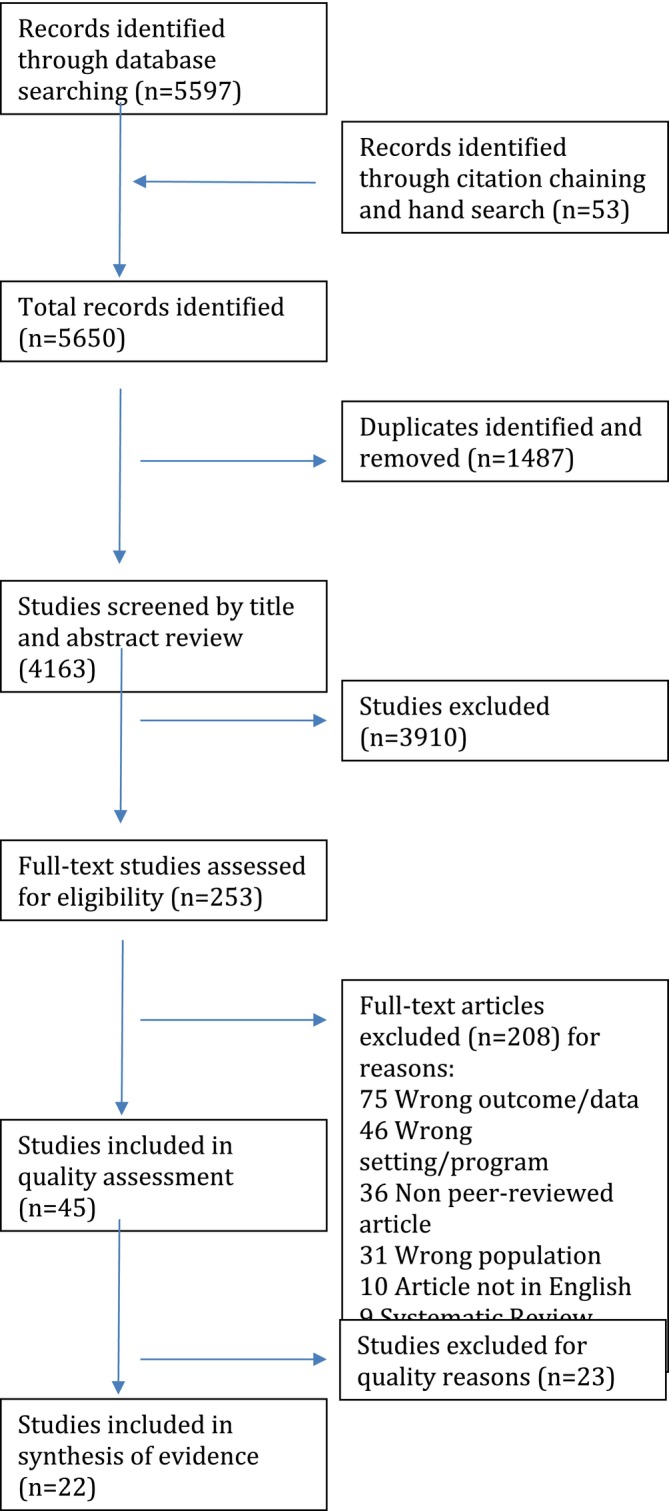
Prisma flow chart.

The search strategy was run on 10 November 2021 and returned 5597 results—469 in Medline, 391 in CINAHL, 1650 in ERIC, 1476 in PsycInfo, 960 in Scopus and 651 in Web of Science. An additional 53 records were identified through hand searches and citation chaining from relevant literature and systematic reviews that were yielded by the search. After the removal of 1487 duplicates using Covidence, 4163 results remained. Of these, 3910 studies were excluded at the stage of title and abstract screening. The full texts of the remaining 253 papers were assessed for eligibility, resulting in the screening out of another 208 studies based on exclusion criteria, which had been ambiguous/absent in the abstracts/titles. This screening phase was conducted by two members of the research team independently (Researchers B and C), with a third research team member (Researcher A) resolving disagreements (see Figure 1 Prisma flow chart).

### 2.2 Quality Assessment

Forty‐five studies were subjected to a thorough quality assessment to ensure only high‐quality evidence informed data analysis. All 45 papers were assessed by at least two members of the research team independently. Disagreements were resolved by a third reviewer or, in a few cases, team discussions. Joanna Briggs Institute (JBI) critical appraisal tools were applied to the 45 papers. The relevant JBI tool (i.e., cohort, cross‐sectional and case study) was selected to match the study design of the paper. The quality assessment resulted in 23 studies being excluded either due to low quality or not fully meeting the inclusion criteria. Subsequently, 22 studies were included in the final review.

Table 2 provides an overview of the 22 papers included in the final review and analysis. All studies were conducted in the global North, with the majority (*n* = 16) being US‐based studies and the remainder comprising three Australian studies and one study each from Ireland, Spain and Iceland.

**TABLE 2:** Summary of included papers.

Study details		Programme details		Participant details				Study design	
References	Country	Programme name and setting	Programme description	Sample size and profile	Primary disability	Additional disabilities	Age and gender	Study type	Data analysis
Agarwal et al. (2021). Evaluating a postsecondary education program for students with intellectual disabilities: Leveraging the parent perspective. Journal of Autism and Developmental Disorders, 51(7), 2229–2240.	USA	Transition & Postsecondary Education Programs for Students with ID (TPSID) nested in a large public university in South Florida	Postsecondary inclusive programme	58 parents of transition‐aged (18–24‐years old) adults with ID who apply and meet basic enrolment criteria for the programme	ID	Autism spectrum disorder (31%), multiple disabilities (8%), speech/language impairments (27%) and other disabilities (34%).	18–24; gender unknown	Qualitative study	Thematic analysis with the coding and theme development guided by the COM‐B model.
Brewer and Movahedazarhouligh (2021). Students with intellectual and developmental disabilities in inclusive higher education: perceptions of stakeholders in a first‐year experience. International Journal of Inclusive Education, 25(9), 993–1009.	USA	Postsecondary Education Program at University in the United States.	A postsecondary inclusive education programme in its first year.	11—4 university teachers with at least one student with ID/DD in class, 4 programme staff members, 3 programme students with ID/DD.	ID/DD		Unknown	Qualitative study.	Thematic analysis through a constructivist paradigm
Casale‐Giannola and Kamens (2006). Inclusion at a university: Experiences of a young woman with Down syndrome. Mental retardation, 44(5), 344–352.	USA	No specific programme. Inclusion of a woman with Down Syndrome in a general speech communications course at a private university.	4‐year speech communications course.	8—young woman included at the college level; her peer support person, her mother; the course instructor; two special education professors who acted as facilitators; non‐disabled peers enrolled in the same college course.	Down Syndrome		21; female (student); other participants unknown	Mixed methods cross‐sectional study. No comparison group.	Thematic data displays.
Cook et al. (2017). Inclusive Concurrent Enrollment: A Promising Postsecondary Transition Practice for Building Self‐Determination among Students with Intellectual Disability. Journal of the American Academy of Special Education Professionals, 25, 44.	USA	Inclusive concurrent enrolment (ICE) at an urban public high school and urban 4‐year university.	Dual enrolment course—students attend high school and audit college course.	9 programme participants (students)	Severe cognitive and/or learning disability (ID).		18–20; six males, three females	Single group cohort study (pretest–posttest design) ‘sequential explanatory design’	Mean survey scores; nonparametric Friedman and Wilcoxon signed‐rank tests; qualitative analysis of interviews using a coding frame consistent with constructs measured in Adolescent Self‐Determination Assessment—Short Form (autonomy, self‐regulation, psychological empowerment and self‐realisation) and inductive review for additional themes.
Hamill (2003). Going to college: The experiences of a young woman with Down syndrome. Mental Retardation, 41(5), 340–353.	USA	Inclusion of a woman with Down Syndrome in a private 4‐year libera larts Jesuit university in the Midwest	A community project programme at the university with a nondegree status, opportunity to audit selected courses and have the tuition waived.	1 student; with DS plus 7 other students (2 study buddies, 3 other classmates and 2 acquaintances in campus settings outside of class); 3 staff members purposively chosen	Down syndrome		26‐year old student with down syndrome; age and genders of others unknown	Ethnographic case study	Coding and thematic analysis of the fieldnotes from the naturalistic observations and from the interviews.
Hendrickson, J. M., Vander Busard, A. M. Y., Rodgers, D., & Scheidecker, B. (2013). College Students with Intellectual Disabilities: How Are They Faring? Journal of College and University Student Housing, 40(1), 186–199.	USA	UI‐REACH Program at University of Iowa	A two‐year certificate (nondegree earning) programme for students with ID.	20 UI‐Reach students, 25 WNS students	No specific disability label		Unknown	Cross‐sectional study	Covariance coefficient representing adjusted mean difference
Hendrickson, J. M., Therrien, W. J., Weeden, D. D., Pascarella, E., & Hosp, J. L. (2015). Engagement among students with intellectual disabilities and first year students: A comparison. Journal of Student Affairs Research and Practice, 52(2), 204–219.	USA	UI‐REACH at University of Iowa	As above	Two groups of UI‐Reach students (*n* = 20 and *n* = 22) Sample size of comparison group unknown	Intellectual and other cognitive disabilities		18–25, mixed genders	Cohort study	Regression‐based analysis of covariance (adjusted mean difference) Statistical controls for several covariates
Lee et al. (2021). Examining growth among college students with intellectual and developmental disability: A longitudinal study. Behaviour Modification, 45(2), 324–348.	USA	Next Steps programme at Vanderbilt (private university)	Inclusive PSE programme	30 students enrolled in programme	Intellectual disability	Autism, learning, ADHD, developmental delay, cerebral palsy and other	18–26‐years old, 1 female, 19 males	Longitudinal study	Descriptive statistics; paired *t*‐tests
Lynch and Getzel (2013). Practice Brief: Assessing Impact of Inclusive Postsecondary Education Using the Think College Standards. Journal of Postsecondary Education and Disability, 26(4), 385–393.	USA	ACE‐IT programme at Virginia Commonwealth University	Access to undergrad courses; fully inclusive	8 enrolled students	Intellectual disability		18–24, 5 females, 3 males	Routine administrative data collection	The think college standards, quality indicators and benchmarks used as framework for evaluation
Moore and Schelling (2015). Postsecondary inclusion for individuals with an intellectual disability and its effects on employment. Journal of Intellectual Disabilities, 19(2), 130–148.	USA	Two programmes—one integrated, one specialised	Integrated programme (audit or credit) and specialised programme compared	2 programme directors, 26 students	Intellectual disability	Autism, dyslexia, visual agnosia, attention deficit hyperactivity disorder	Unknown	Cross‐sectional	Thematic analysis; descriptive statistics
O'Brien et al. (2009). Opening up a whole new world for students with intellectual disabilities within a third level setting. British Journal of Learning Disabilities, 37(4), 285–292.	Ireland	Certificate in Contemporary Living at Trinity College Dublin	Mixed hybrid model, 2‐year certificate programme with audit options with undergrad students for one module.	19 students	Intellectual disability		19 to 48, 13 females, 6 males	Qualitative	Open, axial and selective coding for thematic analysis
Rillotta et al. 2020	Australia	Inclusion of a student with IDD at Edith Cowan University (ECU), Perth, Western Australia	No specific programme, inclusion of student with ID in lectures and tutorial	8—author; student; 6 mentors	Down syndrome		Students age unknown, mentors 19–21 and one older	Qualitative case study	Not stated
Rillotta, F., Arthur, J., Hutchinson, C., & Raghavendra, P. (2020). Inclusive university experience in Australia: Perspectives of students with intellectual disability and their mentors. Journal of Intellectual Disabilities, 24(1), 102–117.	Australia	Up The Hill Program (UTHP)—WIL at Flinders University	Three‐year programme for people with ID to audit six topics at university	4 students with ID and 6 peer mentors	Intellectual disability		19 to 41 (mean ¼ 28 years), two females and two males	Qualitative study	Thematic analysis
Rillotta et al. (2021). The work integrated learning experience of a university student with intellectual disability: a descriptive case study. International Journal of Inclusive Education, 1–18.	Australia	Up The Hill Program (UTHP)—WIL at Flinders University	As above	6—Student with ID, her parents, university and agency supervisors, mentors	Intellectual Disability		Student in her 40s	Descriptive case study, following a realistic evaluation approach	Thematic analysis
Rodríguez Herrero, P., Izuzquiza Gasset, D., & Cabrera Garcia, A. (2021). Inclusive education at a Spanish University: The voice of students with intellectual disability. Disability & Society, 36(3), 376–398.	Spain	Promentor Program—Diploma for Training Young People with Intellectual Disability for Work at Universidad Autonoma de Madrid	2‐year program; main objective is to provide an inclusive university education environment; mixed model of inclusion	14 people who have graduated from the certificate	Intellectual disability		22–44, 6 males, 8 females	Inclusive qualitative study	Content analysis; quant data about each person's participation and number of text segment
Ross et al. (2013). Postsecondary education employment and independent living outcomes of persons with autism and intellectual disability.	USA	Taft College Transition to Independent Living (TIL) programme on Main Campus of a Community College in rural area of Central California	The curriculum consists of 36 individual classes, students receive certificate of completion; and option to enrol in traditional college courses and receive individual supports	125 programme graduates	Intellectual disability		22–37, 70 males (56%) 55 females (44%)	Cross‐sectional study	Descriptive univariate and bivariate statistics, nonparametric tests to determine significance of between group differences; linear regression testing for significant multivariate relationships
Sheppard‐Jones, K., Kleinert, H., Butler, L., & Whaley, B. (2018). Life outcomes and higher education: The need for longitudinal research using a broad range of quality of life indicators. Intellectual and developmental disabilities, 56(1), 69–74.	USA	No specific programme, one of five college campuses in Kentucky participation in State's supported higher education project for students with ID	All coursework was inclusive together with students from general student population.	19 students	Intellectual disability		24.6 average age, 63.2% female	Cross‐sectional study	Frequencies and *t*‐tests
Shogren, K. A., Wehmeyer, M. L., Shaw, L. A., Grigal, M., Hart, D., Smith, F. A., & Khamsi, S. (2018). Predictors of self‐determination in postsecondary education for students with intellectual and developmental disabilities. Education and Training in Autism and Developmental Disabilities, 53(2), 146–159.	USA	Multiple Transition and Postsecondary Programs for Students with Intellectual Disabilities (TPSID) at several colleges and universities	Focus on academic enrichment, socialisation and independent living skills, including self‐advocacy skills; and integrated work experiences and career skills leading to gainful employment	251 programme students	IDD		17–42, 67% (170) males	Cross‐sectional study	Structural equation modelling
Spencer, P., Van Haneghan, J., & Baxter, A. (2021). Exploring social networks, employment and self‐determination outcomes of graduates from a postsecondary program for young adults with an intellectual disability. Journal of Vocational Rehabilitation, 55(3), 251–270.	USA	Comprehensive Transition and Postsecondary (CTP) programme at a University	Inclusive classes mostly with some specialised courses	12 (6 graduates; 6 parents)	Intellectual disability		4 males, 2 females	Cross‐sectional	
Stefánsdóttir and Björnsdóttir (2016 ). ‘I am a college student’ postsecondary education for students with intellectual disabilities. Scandinavian journal of disability research, 18(4), 328–342.	Iceland	Vocational diploma programme at University of Iceland	Vocational diploma programme; in an inclusive setting	39 students; 14 lecturers	Intellectual disability		23–42, Students: 15 M; 24 F Lecturers 8 M; 6 F	Qualitative	Thematic analysis; axial coding
Yuan et al. (2018). From the parents' perspective: The think college experience in rural Vermont. Rural Special Education Quarterly, 37(2), 113–121.	USA	Think College Program at University of Vermont and Johnson State College		9 parents of students with ID	Intellectual disability		Students: 18–30	Qualitative	Transcribed, analysed and coded responses for themes
Zhang et al. (2018). Preparing individuals with disabilities for inclusive employment through the postsecondary access and training in human services (PATHS) program. Inclusion, 6(3), 224–233.	USA	Postsecondary Access and Training in Human Services (PATHS) at Texas A&M University	Provides career training to students with intellectual and developmental disabilities while offering them opportunities to take classes and participate in campus activities with other typical college students.	38 graduates; 6 individual stories (randomly selected)	Intellectual and developmental		4 F; 2 M	Individual stories—narratives	

The studies encompassed a variety of research methods including qualitative (*n* = 8), quantitative (*n* = 6), case study (*n* = 4) and mixed methods (*n* = 4). Study sample sizes ranged from one to 251 participants.

In line with the inclusion criteria, primary disability for the student cohort across studies was intellectual and/or developmental disability, with Down syndrome reported explicitly in two studies. No other aetiologies were reported. Two studies did not include student voice directly but were included because the parent voice focused on student outcomes.

All the studies were of hybrid or full inclusive university programmes as per the criteria for this review, but the type and extent of inclusion differed. The majority of programmes were inclusive classroom and campus models, with students participating, along with their peers without intellectual disability, in lessons, social activities and university life. Two hybrid models were included for the inclusive elements they afforded students via the auditing of classes of their choice with peers. Three programmes focused on inclusion in specific disciplines of study—education (*n* = 2) and speech communication (*n* = 1). One programme reported on students participating in an inclusive university programme while also studying some units in high school.

### 2.3 Data Extraction

To systematically review the 22 studies meeting the inclusion criteria and passing the quality assessment, a data extraction table was created. Along with the study details (country; sample size, method and profile; study design; measures used; outcomes measured), summarised above, the data extracted from the studies also included all study findings answering our research questions about student outcomes (autonomy, self‐confidence, social inclusion, social skills and employment) and facilitators and barriers to these outcomes. Categories used for coding of the facilitators and barriers were developed inductively. An ‘other’ category was included in the data extraction table to capture any additional outcomes reported, which may not have been identified in the initial scoping search. The data extraction of the final 22 papers was conducted by four researchers, with two reviewers performing data extraction for each article separately before cross‐checking with one another to ensure inter‐rater consistency. Team meetings took place to review any conflicts and to ensure overall agreement within the team.

### 2.4 Data Synthesis

A thematic synthesis of the qualitative findings (Thomas and Harden 2008) was developed by Researcher A. The initial thematic analysis was reviewed, debated and then refined through discussion and agreement by all three authors. This approach was used for the thematic analysis and synthesis of outcomes as well as data related to facilitators and barriers. Though not all papers were purely qualitative, there was limited quantitative data (*n* = 9), likely due to the research being mostly about lived experience; thus, no meta‐analysis was possible. However, some descriptive statistics from the papers are reported where applicable. In addition, where standardised measures were used within longitudinal or pre‐/post‐test or comparative cohort studies, this is highlighted.

## 3 Findings

The synthesised findings answering our research questions are presented here as key themes under each of the broader themes of student outcomes and the facilitators and barriers to student outcomes. They are also summarised by study in Table 3.

**TABLE 3:** Barriers, facilitators and outcomes by study.

Study details		Programme details		Outcomes					Barriers and facilitators	
References	Country	Programme name and setting	Autonomy	Self‐confidence	Social inclusion	Employment	Social skills	Other	Barriers	Facilitators
Agarwal et al. (2021). Evaluating a postsecondary education program for students with intellectual disabilities: Leveraging the parent perspective. Journal of Autism and Developmental Disorders, 51(7), 2229–2240.	USA	Transition & Postsecondary Education Programs for Students with ID (TPSID) nested in a large public university in South Florida	***✔***	***✔***	***✔***	***✔***		***✔***	Some skill areas remained underdeveloped. Parents overprotectiveness was a barrier to development	Immersion in college environment
Brewer and Movahedazarhouligh (2021). Students with intellectual and developmental disabilities in inclusive higher education: perceptions of stakeholders in a first‐year experience. International Journal of Inclusive Education, 25(9), 993–1009.	USA	Postsecondary Education Program at University in the United States.	***✔***		***✔***	***✔***		***✔***	No transition prep for students or parents (letting go). Students not having clear understanding of expectations.	Staff understanding one another's roles and having time to plan and access to training for staff
Casale‐Giannola and Kamens (2006). Inclusion at a university: Experiences of a young woman with Down syndrome. Mental retardation, 44(5), 344–352.	USA	No specific programme. Inclusion of a woman with Down syndrome in a general speech communications course at a private university.	***✔***		***✔***		***✔***	***✔***	Large class sizes. Transport availability. Commuter students found it difficult to engage in nonclass activities (other aspects of college life). Inconsistent understanding of goal setting. Lack of skill/experience among teaching staff re: marking	Being interested in working with people with IDD Willingness of academic staff to include people with IDD in their classes and make adaptations. Peer support; Extensive coordination, flexibility and support; review of goals
Cook et al. (2017). Inclusive Concurrent Enrollment: A Promising Postsecondary Transition Practice for Building Self‐Determination among Students with Intellectual Disability. Journal of the American Academy of Special Education Professionals, 25, 44.	USA	Inclusive concurrent enrolment (ICE) at an urban public high school and urban 4‐year university.	***✔***					***✔***		
Hamill (2003). Going to college: The experiences of a young woman with Down syndrome. Mental Retardation, 41(5), 340–353.	USA	Inclusion of a woman with Down syndrome in a private 4‐year liberal arts Jesuit university in the Midwest			***✔***	***✔***	***✔***	***✔***	Relying on student to initiate engagement in class. Study buddies did not always feel competent to support. Not receiving grades was a barrier to the student feeling part of the full uni experience	Study buddies
Hendrickson, J. M., Vander Busard, A. M. Y., Rodgers, D., & Scheidecker, B. (2013). College Students with Intellectual Disabilities: How Are They Faring? Journal of College and University Student Housing, 40(1), 186–199.	USA	UI‐REACH Program at University of Iowa	***✔***					***✔***		Integrated residence halls with support from residence hall assistants
Hendrickson, J. M., Therrien, W. J., Weeden, D. D., Pascarella, E., & Hosp, J. L. (2015). Engagement among students with intellectual disabilities and first year students: A comparison. Journal of Student Affairs Research and Practice, 52(2), 204–219.	USA	UI‐REACH at University of Iowa			***✔***			***✔***		
Lee et al. (2021). Examining growth among college students with intellectual and developmental disability: A longitudinal study. Behaviour Modification, 45(2), 324–348.	USA	Next Steps programme at Vanderbilt (private university)	***✔***				***✔***	***✔***		
Lynch and Getzel (2013). Practice Brief: Assessing Impact of Inclusive Postsecondary Education Using the Think College Standards. Journal of Postsecondary Education and Disability, 26(4), 385–393.	USA	ACE‐IT programme at Virginia Commonwealth University	***✔***	***✔***		***✔***		***✔***		
Moore and Schelling (2015). Postsecondary inclusion for individuals with an intellectual disability and its effects on employment. Journal of Intellectual Disabilities, 19(2), 130–148.	USA	Two programmes—one integrated, one specialised	***dk***	***dk***	***dk***	***✔***	***✔***			
O'Brien et al. (2009). Opening up a whole new world for students with intellectual disabilities within a third level setting. British Journal of Learning Disabilities, 37(4), 285–292.	Ireland	Certificate in Contemporary Living at Trinity College Dublin	***✔***	***✔***	***✔***	***✔***	***✔***	***✔***		
O'Rourke (2011). Inclusion at University: Can we do more than open the door?	Australia	Inclusion of a student with IDD at Edith Cowan University (ECU), Perth, Western Australia	***✔***	***✔***	***✔***	***Nil***	***✔***	***✔***	Negative attitudes and perceptions at different levels of Uni with regard to place of people with ID in Uni. Not treated as a full student as not enrolled.	Support from Uni leadership
Rillotta et al. (2020). Inclusive university experience in Australia: Perspectives of students with intellectual disability and their mentors. Journal of Intellectual Disabilities, 24(1), 102–117.	Australia	Up The Hill Program (UTHP)—WIL at Flinders University	***✔***	***✔***	***✔***	***✔***		***✔***	Student peers tendency to speak to mentors of student rather than student with ID	Training for lecturers
Rillotta et al. (2021). The work integrated learning experience of a university student with intellectual disability: a descriptive case study. International Journal of Inclusive Education, 1–18.	Australia	Up The Hill Program (UTHP)—WIL at Flinders University	***✔***		***✔***		***✔***	***✔***	Social beliefs about disability	
Rodríguez Herrero, P., Izuzquiza Gasset, D., & Cabrera Garcia, A. (2021). Inclusive education at a Spanish University: The voice of students with intellectual disability. Disability & Society, 36(3), 376–398.	Spain	Promentor Program—Diploma for Training Young People with Intellectual Disability for Work at Universidad Autonoma de Madrid		***✔***	***✔***	***✔***	***✔***	***✔***	Inaccessible environment. Personal anxiety. Rigid teaching approaches	Accepting lecturers and access to specialist tutor. Not being treated as different; family clear structured modules
Ross et al. (2013). Postsecondary education employment and independent living outcomes of persons with autism and intellectual disability.	USA	Taft College Transition to Independent Living (TIL) programme on Main Campus of a Community College in rural area of Central California	***✔***	***✔***		***✔***	***✔***	***✔***		
Sheppard‐Jones, K., Kleinert, H., Butler, L., & Whaley, B. (2018). Life outcomes and higher education: The need for longitudinal research using a broad range of quality of life indicators. Intellectual and developmental disabilities, 56(1), 69–74.	USA	No specific programme, one of five college campuses in Kentucky participating in State's supported higher education project for students with ID			***✔***	***✔***	***✔***	***✔***		
Shogren, K. A., Wehmeyer, M. L., Shaw, L. A., Grigal, M., Hart, D., Smith, F. A., & Khamsi, S. (2018). Predictors of self‐determination in postsecondary education for students with intellectual and developmental disabilities. Education and Training in Autism and Developmental Disabilities, 53(2), 146–159.	USA	Multiple Transition and Postsecondary Programs for Students with Intellectual Disabilities (TPSID) at several colleges and universities	***✔***							
Spencer, P., Van Haneghan, J., & Baxter, A. (2021). Exploring social networks, employment and self‐determination outcomes of graduates from a postsecondary program for young adults with an intellectual disability. Journal of Vocational Rehabilitation, 55(3), 251–270.	USA	Comprehensive Transition and Postsecondary (CTP) programme at a University	***✔***		***✔***	***✔***			Impact on disability benefits—fear. Staff turnover and knowledge gaps	Family and mentor support
Stefánsdóttir and Björnsdóttir (2016). ‘I am a college student’ postsecondary education for students with intellectual disabilities. Scandinavian journal of disability research, 18(4), 328–342.	Iceland	Vocational diploma programme at University of Iceland		***✔***	***✔***	***✔***	***✔***	***✔***	Limited focus of course within one discipline. Limited to 2 years and no opportunity for further study. Negative perceptions by some staff re: place of students on campus	Positive attitudes from lecturers and other students
Yuan et al. (2018). From the parents' perspective: The think college experience in rural Vermont. Rural Special Education Quarterly, 37(2), 113–121.	USA	Think College Program at University of Vermont and Johnson State College	***✔***	***✔***		***✔***	***✔***		Transport. Parental fears. Lack of residential options. Costs	Professor comfort level increasing; support from educators and mentors; parents support
Zhang et al. (2018). Preparing individuals with disabilities for inclusive employment through the postsecondary access and training in human services (PATHS) program. Inclusion, 6(3), 224–233.	USA	Postsecondary Access and Training in Human Services (PATHS) at Texas A&M University	***✔***	***✔***	***✔***	***✔***			Navigating college space. Money management	Assistive technology. Tutors. Practical experience

### 3.1 Student Outcomes

The studies showed that participation in inclusive university programmes results in increased autonomy, self‐confidence, employment and social skills.

#### 3.1.1 Autonomy

Evidence of greater autonomy and control as a result of participation in inclusive programmes (Brewer and Movahedazarhouligh 2021) included increased independent living skills (Agarwal et al. 2021; Lynch and Getzel 2013), independent travel (O'Rourke 2011), independent learning/studying (Agarwal et al. 2021), better problem‐solving skills (Lynch and Getzel 2013), increased decision‐making skills (Brewer and Movahedazarhouligh 2021; Casale‐Giannola and Kamens 2006) and greater self‐determination (Lee et al. 2021; Lynch and Getzel 2013). Specifically, Lee et al. (2021) found an increase in self‐determination using the ARC self‐determination scale, between freshman and second year. One study found that some students were participating more in their communities, such as getting involved in church activities or voluntary work (Agarwal et al. 2021). However, another study found that, while students participating in a programme for two or three semesters had demonstrated growth in self‐determination, no growth in self‐determination was evident for students who only attended for one semester based on mean self‐determination score (Cook et al. 2017). Shogren et al. (2018) reported that access to one additional social activity had a positive impact on autonomy (again using the ARC self‐determination scale). One area where self‐determination was not found to improve was in employment choice (Spencer et al. 2021). This was despite overall improvement in self‐determination. Hendrickson et al. (2013) used the Ryffs Scale of Psychological well‐being, which includes an indicator of autonomy, but reported no statistically significant difference between cohorts.

#### 3.1.2 Self‐Confidence

Increased self‐confidence of programme participants was reported almost universally across the papers reviewed. This was demonstrated through students taking leadership roles and through their heightened comfort working with colleagues, developing and delivering presentations (Casale‐Giannola and Kamens 2006), their confidence in their abilities (Cook et al. 2017), in speaking up (O'Brien et al. 2009) and in working towards goals and taking responsibility (Agarwal et al. 2021). Reports of boosted self‐esteem (Stefánsdóttir and Björnsdóttir 2016), sense of pride (Cook et al. 2017; Rodríguez Herrero et al. 2021), sharing aspirations (O'Rourke 2011) and ‘walking with head up’ (Yuan et al. 2018) were other indicators of increased confidence. Some students became more aware of their rights (Stefánsdóttir and Björnsdóttir 2016).

#### 3.1.3 Employment

Positive employment outcomes were reported for inclusive programmes where the goal of employment was a major focus of the programme (Lynch and Getzel 2013; Moore and Schelling 2015; Stefánsdóttir and Björnsdóttir 2016; Yuan et al. 2018). Postprogramme employment levels reached 82%–84% in two of the studies (Rodríguez Herrero et al. 2021; Ross et al. 2013). Work placements were found to be a useful tool in leading to employment (O'Brien et al. 2009). However, there was no guarantee of permanent work, and some parents were fearful that students would lose welfare payments if employed, and subsequently were less inclined to support employment opportunities (Yuan et al. 2018). One study showed that employment type was influenced by programme type, with graduates who attended segregated programmes being more likely to work in blue‐collar jobs like food handling or janitorial work, while office work was more likely to be an option for graduates who participated in an integrated/inclusive programme (Moore and Schelling 2015).

#### 3.1.4 Social Skills

There was a noted improvement in social skills among programme participants, demonstrated through public speaking, expressing feelings, introducing self to class, recognising others' points of view and engaging in advocacy (Agarwal et al. 2021; O'Rourke 2011; Stefánsdóttir and Björnsdóttir 2016). There is also evidence that these skills were transferred to other contexts. For instance, overcoming shyness and improving overall communication skills on campus enabled some students to engage with workmates on placement (Rodríguez Herrero et al. 2021). However, one study found no significant changes in social skills among students, using the Social Skills Improvement System Rating Scale, though that may have been due to the students already having good social skills at the time they entered the programme (Lee et al. 2021).

Despite improvements in social skills, the impact of the programmes on social inclusion was more variable. Some students reported making friends and feeling comfortable with the new environment, with other students and with workmates (Lynch and Getzel 2013) and/or feeling valued and accepted in the university (Rodríguez Herrero et al. 2021). However, there were also reports of difficulties initiating interactions (Hamill 2003) and of problems maintaining social relationships (O'Rourke 2011). Though there was increased frequency of interactions between students and mentors, this did not always extend beyond the programme into meaningful relationships and friendships (Casale‐Giannola and Kamens 2006). When asked about friendships, students often spoke of other students in the inclusive programme rather than the wider student cohort (Brewer and Movahedazarhouligh 2021).

#### 3.1.5 Other Outcomes

Other outcomes not included as focus areas in the review include increases in knowledge, academic skills and learning (Agarwal et al. 2021; Hamill 2003); self‐identity and pride (Cook et al. 2017; Hamill 2003), community living (Ross et al. 2013) and engaging in behaviours typical of peers (O'Rourke 2011). Adaptive behaviour (using Vineland Adaptive Behaviour Scale) was found to improve between two time points on some domains, but no change in executive functioning (as measured through the Brief Executive Functioning test; Lee et al. 2021). Hendrickson et al. (2013) used the Openness to Diversity/Change scale to measure openness to diversity but was not found to be statistically different.

Though many of the inclusive programmes did not require students to complete assignments or give credit for work completed, academic learning was widely reported (O'Brien et al. 2009). There was increased topic knowledge (Rillotta et al. 2020), broadening of academic interests (Lynch and Getzel 2013) and improved reading and writing skills (Agarwal et al. 2021). Learning occurred across the curriculum (Rodríguez Herrero et al. 2021), as well as across groups, with mutual learning for teachers, parents, classmates peer support person (Casale‐Giannola and Kamens 2006) and mentors (Agarwal et al. 2021; Hamill 2003). Some students wanted the opportunity to engage in credit‐bearing learning, while others had no desire to complete academic work and were primarily interested in the social aspects of university life (Brewer and Movahedazarhouligh 2021). There were also reports of some students struggling with academic work and of it creating anxiety (Brewer and Movahedazarhouligh 2021; Hamill 2003; O'Rourke 2011).

Another finding outside the focus of our review was the benefits of the inclusion of students with intellectual disability to staff (Stefánsdóttir and Björnsdóttir 2016), mentors/peers (O'Rourke 2011) and families as a result of the students' participation, learning and increased independence (Agarwal et al. 2021).

### 3.2 Facilitators and Barriers to Achieving Positive Outcomes

Participants across the studies identified a range of factors that facilitated or impeded access to, participation in and/or positive outcomes from university for students with intellectual disability. These are described under six thematic areas—accessibility, expectations, supports, attitudes and acceptance, pedagogy and programme design.

#### 3.2.1 Accessibility

Physical barriers to university included campus inaccessibility and complexity. Students who had physical, as well as intellectual, disabilities found some classrooms or campuses physically inaccessible (Rodríguez Herrero et al. 2021). Other students reported difficulties finding their way around campus, like the student who likened the complexity of campus to a labyrinth (Rodríguez Herrero et al. 2021). Nevertheless, students also benefitted from the process of having to find their way independently to and around campus, gaining independent living skills in the process (Agarwal et al. 2021). One group of students who had been provided transport to attend high school because they lived in a rural area was not provided any transport to attend college, and encountered difficulties getting to campus (Yuan et al. 2018). Having to commute to campus made it difficult for non‐residential students to engage in some aspects of college life, making the non‐academic activities less accessible (Yuan et al. 2018).

Accessibility also included financial accessibility. The parents of some students living in a rural area with no public transport could not afford to keep driving their child the long distance to college (Yuan et al. 2018).

#### 3.2.2 Expectations

Low expectations by the university community as a whole resulted in some students not being considered as, or treated like, a ‘real’ student. For example, being audited into classes rather than being fully enrolled (O'Rourke 2011) and not receiving grades or being required to complete or pass assessments (Hamill 2003) were perceived by some students as barriers to genuine inclusion. Being restricted to one discipline (Stefánsdóttir and Björnsdóttir 2016) or given a limited choice of electives was also perceived as an obstacle to authentic inclusion (Stefánsdóttir and Björnsdóttir 2016) by some students.

Some staff held reservations about the academic capacities of students with intellectual disability (Stefánsdóttir and Björnsdóttir 2016). The fears, overprotectiveness and uncertainties of some parents of students with intellectual disability, such as doubting their adult child's ability to engage with the academic content or to develop independent living skills, could also be a barrier to flourishing at university (Agarwal et al. 2021; Zhang et al. 2018). Indeed, Agarwal et al. (2021, 2234) found that parental expectations and attitudes were key to shaping their child's inclusive programme experiences.

#### 3.2.3 Supports

Support from university staff was found to be important for positive student experiences and the success of inclusive programmes (O'Rourke 2011; O'Brien et al. 2009). Mentors and families, in particular parents, played a crucial support role for the students (Agarwal et al. 2021; O'Brien et al. 2009; Spencer et al. 2021). Supports that were natural (e.g., unpaid/informal) and collaborative worked well to facilitate social inclusion (Hamill 2003) and increase employment connections (Spencer et al. 2021) as well as to help others to understand inclusion and encourage a mindset that inclusion works (Brewer and Movahedazarhouligh 2021). Peer mentoring is a common and highly successful example of this. However, the support of specialist teacher‐tutors was also valued by students (Rodríguez Herrero et al. 2021). So too was the support and encouragement of families (Rodríguez Herrero et al. 2021), particularly parents (Agarwal et al. 2021; Yuan et al. 2018). In one programme, academic support in the form of iPads and public speaking classes was found to be helpful (Agarwal et al. 2021).

Given that support was found to be a key enabler, it is unsurprising that inadequate levels of support were found to be a barrier to inclusion and achieving programme outcomes (Stefánsdóttir and Björnsdóttir 2016). However, poor support from teaching staff may be attributed to a lack of training and support (Brewer and Movahedazarhouligh 2021), in particular training on how to address the uncertainty and resistance to grade work (Casale‐Giannola and Kamens 2006) and can also be due to a lack of time (Casale‐Giannola and Kamens 2006) and, in one university, staff turnover (Spencer et al. 2021). One programme struggled to get the balance between support and facilitating independence right for all students (Yuan et al. 2018). In some programmes, a potential over‐reliance on mentors was found to be problematic (O'Rourke 2011; Yuan et al. 2018).

#### 3.2.4 Attitudes and Acceptance

Unsurprisingly, the acceptance of, and positive attitudes towards, students with intellectual disabilities both in the classroom and beyond were found to be key to their successful inclusion (Rodríguez Herrero et al. 2021). Other students having a positive attitude towards students with intellectual disability and not treating them as different were found to be particularly helpful in facilitating inclusion (Rodríguez Herrero et al. 2021). University teachers who are at ease in working with students with intellectual disability (Lynch and Getzel 2013) and willing to adapt their teaching to the needs of students with intellectual disability fostered an inclusive classroom environment (O'Rourke 2011; O'Brien et al. 2009; Rodríguez Herrero et al. 2021).

Conversely, negative attitudes of staff and other students impeded successful programme outcomes. For example, some of the negative attitudes and perceptions included ableist and elitist attitudes of university management (O'Rourke 2011; Stefánsdóttir and Björnsdóttir 2016), infantilisation by some teachers (Stefánsdóttir and Björnsdóttir 2016) and a failure by mentors and other students to extend inclusion beyond the context of the programme (Casale‐Giannola and Kamens 2006; Hamill 2003). From a parent's perspective, negative attitudes regarding the impact of participating in inclusive university programmes on access to disability benefits resulted in some adult children being discouraged from participation (Spencer et al. 2021).

#### 3.2.5 Pedagogy

Pedagogical facilitators of inclusion and positive outcomes reported include access to specialist tutors (Rodríguez Herrero et al. 2021), use of student‐centred teaching approaches (Rodríguez Herrero et al. 2021), clearly structured modules (Rodríguez Herrero et al. 2021) and small class sizes (Casale‐Giannola and Kamens 2006; Hamill 2003). Adapting teaching to the learning styles of students, for example, replacing written instructions with visual or hands‐on instructions and using repetition and consistency in the delivery of information, was found to better facilitate learning for students with intellectual disabilities (Agarwal et al. 2021).

Pedagogical barriers reported include teaching staff not using learner‐centred methods, large class sizes prohibiting peer interaction (Casale‐Giannola and Kamens 2006) and teachers not having the training, support nor the additional time required to support students (Brewer and Movahedazarhouligh 2021; Casale‐Giannola and Kamens 2006; Hamill 2003; Stefánsdóttir and Björnsdóttir 2016).

#### 3.2.6 Programme Design

A well‐designed programme was also important in facilitating successful programme outcomes. According to the papers reviewed, this includes having clear programme objectives (Moore and Schelling 2015), ensuring compatibility and good, open communication between all stakeholders involved in the programme (Brewer and Movahedazarhouligh 2021), building in a review of goals and progress for the students during the programme (Casale‐Giannola and Kamens 2006), preparing students for work experience before any placement occurs (Rillotta et al. 2021) or preparing students for a chosen career postgraduation (Zhang et al. 2018) including planned, as well as natural, opportunities for socialising both on and off campus (Agarwal et al. 2021). Screening during enrolment for students most likely to benefit from and succeed in the programme (Lee et al. 2021; Moore and Schelling 2015; Rodríguez Herrero et al. 2021) was also important. Along with academic pre‐requisites, student characteristics found to facilitate positive programme outcomes were self‐determination, attention to detail, organisational skills, confidence, independence and persistence (Rillotta et al. 2021).

Non‐academic programme components beneficial to students include participation in extracurricular activities/clubs (Agarwal et al. 2021; Hamill 2003), being taught independent living skills (Agarwal et al. 2021), employment (Agarwal et al. 2021; Zhang et al. 2018) and advocacy skills (Stefánsdóttir and Björnsdóttir 2016; Zhang et al. 2018). An additional benefit is the option to live in residential halls, which parents in one programme stated had ‘contributed significantly to student growth’ (Agarwal et al. 2021, 2233).

Some of the barriers to positive programme outcomes reported were lack of communication, collaboration and delineation of clear roles and responsibilities among programme staff (Brewer and Movahedazarhouligh 2021); poor pre‐planning and preparation (Brewer and Movahedazarhouligh 2021); absence of a pre‐enrolment ‘readiness program’ for students or the enrolment of students not yet ready to attend university (Brewer and Movahedazarhouligh 2021); not preparing parents for their child's transition to university (Brewer and Movahedazarhouligh 2021); lack of dedicated funding/resources for the inclusion of students with intellectual disability (Casale‐Giannola and Kamens 2006); poor communication with students about university requirements and expectations (Brewer and Movahedazarhouligh 2021); and inconsistency of goals and expectations among stakeholders, i.e., the student, peers, instructors and parents (Casale‐Giannola and Kamens 2006); one example being lack of clarity for ‘study buddies’ about the extent of support they should provide (Hamill 2003).

## 4 Discussion

Access to and participation in higher education and specifically university has many known benefits. These include improved health, quality of life, self‐esteem and confidence. In addition, increased social connections and opportunities, including increased opportunities for employment, which in turn have societal economic benefits, have been found to materialise. The benefits are multifaceted and impact directly on the individual, their family and society. People with intellectual disability have traditionally not had the opportunity to attend university and have subsequently been denied access to the associated benefits. The increase in specialised programmes and pathways internationally is providing the chance for more people with intellectual disability to benefit in these ways.

The current review confirms earlier indications (Grigal et al. 2018) that participation in inclusive university programmes leads to primarily positive outcomes for people with intellectual disability. Much has already been written about the impact of these programmes on employment outcomes for people with intellectual disability. As anticipated, where employment was a core focus for a program, with work placement built into the structure, high rates of employment resulted upon completion. Given that 16 of the 22 articles involved American programmes, this may reflect the funding arrangements in the United States, coupled with the focus of inclusive programs in America on employment outcomes (Grigal et al. 2022). Given that people with intellectual disability are less likely to be in open employment compared with non‐disabled people and other people with disability (Almalky 2020), the positive employment outcomes due to participation in university reported in the reviewed studies are welcome and are one mechanism to support the move of people with intellectual disability out of poverty, of which they are one of the greatest groups at risk (Emerson 2007). Nevertheless, permanent employment did not always follow (Yuan et al. 2018), and there was limited discussion of career development in the papers reviewed. There was also limited indication of what happens over time if the person wishes to change roles or employer, and the extent to which the initial employment experience aids this.

The present review highlights the range of positive outcomes feasible in addition to employment. There was noted improvement in confidence and self‐determination, social skills, engagement and responsibility as well as increased social interactions among programme participants. Expanding social networks and building social capital are important outcomes for the general student population. The opportunity to build greater networks of friends through university participation has added importance for people with intellectual disability due to typically smaller social networks. In addition, social inclusion is an important nonmedical determinant of health (Perez et al. 2022). However, there is a question over the meaningfulness and sustainability of the relationships that students with intellectual disability formed with students without disability and the extent to which they engaged in socialising beyond university. This is concerning yet indicative of other research. Matheson et al. (2007) reported greater stability in friendships between young people with intellectual and developmental disability, compared with friendships between these young people and peers without disability.

Though attaining credit was not available to most students in these studies, learning both academic and content knowledge was achieved. There was also mutual learning for other stakeholders involved in the programmes. While most students wished to engage in academic learning, some students preferred engagement in the social side of university life.

Of importance for future practice is understanding the barriers and facilitators to successful participation in inclusive programmes and to the achievement of positive outcomes. To ensure success in the operation of, and participation in, an inclusive university program, the evidence indicates that a range of supports is required. This involves top‐down support from university leadership (O'Rourke 2011) as well as natural and collaborative supports from family and mentors. For teachers, a willingness and ability to adjust teaching and learning approaches as well as assessment style and grading is essential. Arguably, this is within legislative requirements and moral obligations (O'Donovan 2021). However, whether this is possible in practice depends on funding, resources, training and the support of university management. Universities seeking to offer inclusive programmes for students with intellectual disability should consider these barriers and facilitators in the planning of program design.

Beyond the classroom, non‐academic activities and opportunities enhance learning experiences and further support the achievement of outcomes. This is a well‐known indicator of success for university students in general (Karp 2011), and therefore also applicable for students with intellectual disability.

Families have also reported that university experience raises the bar by setting an expectation to achieve that did not previously exist (O'Brien and Murray 2019). Inclusive university can also increase students' expectations of themselves by challenging and stimulating them to achieve (Rillotta et al. 2019).

Bonati (2019) noted that perceptions and expectations held by lecturers were impacted positively by the fact that students with intellectual disability had great attendance, were not afraid to ask questions and demonstrated best efforts to learn. However, despite decreasing, negative attitudes and perceptions towards the inclusion of students with intellectual disability in the university campus persist. There remains resistance to accommodate and adapt, as well as resistance to grading assessments and providing opportunities for credit‐bearing engagement.

Despite these challenges, the positive outcomes that can result from participation in university life, both academically and socially, must not be underestimated. These benefits extend beyond the student with intellectual disability to other students, faculty, family members and society. There are also likely economic benefits due to greater employment and independence achieved. Though economic analysis was not the focus of any paper, the review demonstrates the potential benefits, but much work is needed to progress the development of, and access to, inclusive programmes internationally. Most of the papers reviewed were American, with some Australian and European programmes represented. This reflects the specific policy and legislative structures in place with the United States to support these initiatives, which are not comparable elsewhere. Higher education policy at the national level should reflect the specific needs of, and raise the bar for, people with intellectual disabilities.

## 5 Limitations and Future Directions

This review has some limitations. Programmes that did not incorporate an inclusive element were not included in this review. Thus, papers that reported on outcomes from segregated models were missed. The review was limited to English‐only articles. The authors are aware of programmes in non‐English speaking countries in Europe and the global South, and findings from these programs would have been missed if not published in English. This review was limited to academic peer‐reviewed literature. Books, book chapters, unpublished dissertations and grey literature documents were excluded, and as such, additional insights into outcomes may be missed. Methodologically, the small sample sizes achieved in most of the studies reviewed limit any generalisation being made. The limited longitudinal data means that change over time and longer‐term impacts on students are not known.

In the process of conducting our literature review, we found that many inclusive programmes, including credit‐attaining programs with a qualification, have either not conducted research on their programmes, conducted research but not published their findings, or published findings, which were ineligible for our review due to not meeting quality appraisal criteria. Though likely due primarily to limited resources, this is problematic as it means lessons learned are not as widely shared as they could be, resulting in potential unnecessary duplication and repetition across programmes. It may also serve to perpetuate barriers to successful outcomes. Only one of the papers we reviewed compared models of inclusion. Moore and Schelling (2015) compared the impact of the programme model on employment outcomes. This lack of comparative studies makes it difficult to say which model is more inclusive, and hence to make recommendations for best practice.

Our review suggests several clear directions/areas of focus for implementing and researching inclusive programmes. Firstly, there is clearly a need for inclusive programmes (and research) to thoroughly consider the meaningfulness and quality of relationships formed by programme participants with students without disability and to explore strategies to nurture more meaningful relationships and promote socialising beyond university. Secondly, as inclusive education in many jurisdictions is no longer in the emergent phase, it is time for inclusive university programmes to meet the increasing need/desire for accredited inclusive programmes, alongside more programmes focused on socio‐emotional outcomes for students who may prefer that option. Thirdly, as more and more inclusive programmes are being established globally, there is an urgent need for clearer evidence about which inclusive programme models and/or aspects of inclusive programmes are linked to which student outcomes. There is also a need to share resourcing, learning and approaches on design and implementation that enhance those facilitators and minimise the barriers identified. Implementation of programmes needs to be culturally appropriate to maximise potential for success and sustainability. Thus, it is imperative for existing inclusive programmes to conduct research evaluating outcomes aligned with their programme's objectives and characteristics, to measure outcomes over time, to publish that research and for that research to be high‐quality research.

## 6 Conclusion

Our review reinforces well‐established positive impacts of inclusive programmes on the lives of students with intellectual disability and highlights the breadth of positive outcomes that are possible, particularly in terms of increased autonomy, self‐confidence, social inclusion, social skills and employment. By identifying specific barriers and facilitators to these positive outcomes for participants in inclusive programmes, our review also provides an up‐to‐date overview of indicators of best practice for the inclusion of people living with intellectual disability in university.

What we already know is that inclusive programmes require a whole‐of‐university approach to supporting full and genuine inclusion of students with intellectual disability from enrolment through to graduation and beyond. This includes support in getting to and navigating around campus, physically, socially and administratively; an accepting and inclusive university‐wide culture, which means having an option to enrol as a fully‐fledged student but also goes beyond the classroom and into residential halls, student clubs and other non‐academic aspects of university life, engendering a sense of belonging; student‐centred teaching with wrap‐around (preferably natural) support from teachers, students and families. Holistic and authentic inclusion of students with intellectual disability in university will benefit all students and lead to a more diverse campus. Inclusive programmes need to include work experience and work placement to ensure the pathway for students with intellectual disability does not stop abruptly after graduation.

Outside of the higher education sector, continued advocacy of the rights of people with intellectual disability to postsecondary school options, including university, is required (Wedgwood et al., accepted). This may mean advocating to the government in countries that provide no or only limited access to university for their citizens with intellectual disability, despite being signatories to the Convention of the Rights of Persons with Disability (CRPD), which enshrines the right to access tertiary education without discrimination and *on an equal basis with others* (United Nations 2008: Article 24.5).

## Ethics Statement

This was a desk‐based rapid review of literature. Therefore, no ethical approval was required for this study. Ethical practice was a core part of the process in particular, the quality assessment of included papers.

## Conflicts of Interest

The authors declare no conflicts of interest.

## Data Availability

A sample search string is provided in the paper. The authors can make all search strings for each database available upon request so that the search can be replicated, and other researchers can access the same data.
